# Dental Law in Michigan

**Published:** 1883-08

**Authors:** 


### Dental Law in Michigan.

In conformity to the law regulating the practice of dentistry in Michigan, the Governor has appointed a Board of Examiners, consisting of Dr. A. T. Metcalf, of Kalamazoo, for a term of one year, President; Dr. J. A. Robinson, of Jackson, for a term of two years, Treasurer; and Dr. G. R. Thomas, of Detroit, for a term of three years, Secretary.

The appointment of these gentlemen as members of this Board will certainly be acceptable to the profession of Michigan, and it is to be hoped, to the gentlemen themselves, though they will find a large amount of hard work.

The profession of this State should note the law closely, in which they will find that it is necessary for every practitioner of dentistry in the State to send an affidavit to the Secretary of said Board, in which he shall give his name, place of business, post office address, and length of time he has been engaged in practice in this State, and if he is a graduate of a dental college, state the name of the same, and also pay the Treasurer of said Board the sum of twenty-five cents, and if he fails to comply with this request, he must appear and be examined by said Board. This is an excellent requirement, and is one step in advance of most of the laws on this subject. It will be to the interest of every dentist in the State to see that the law is sustained and its requirments obeyed. It would certainly be unjust for a part to conform to it and others be allowed to go free or violate the spirit or letter of the law. The profession of Michigan should be congratulated for the excellent law which they have attained, as well as for the competent and energetic Board of Examiners. Michigan, from this time forth, ceases to be the eldorado of quacks. For years past the imigration from surrounding States to Michigan has been large, consisting mainly of those who found it inconvenient to practice dentistry in neighboring States having restriction laws. In this way, those who are now practicing, cannot be molested, but the increase of incompetent dentists will be absolutely stopped and prevented if the law is faithfully executed. The duty devolves upon the better part of the profession in the State toward those who are incompetent but honest, to induce them to increase their professional knowledge and ability. This is a work to be done mainly by the individuals of the profession throughout the State. Let every good dentist help his weak brother so far as he can. The revolution will not be wrought at once, but a few years will bring a very great change.

